# Soil Moisture Availability at Early Growth Stages Strongly Affected Root Growth of *Bothriochloa ischaemum* When Mixed With *Lespedeza davurica*

**DOI:** 10.3389/fpls.2018.01050

**Published:** 2018-08-06

**Authors:** Zhi Wang, Weizhou Xu, Zhifei Chen, Zhao Jia, Jin Huang, Zhongming Wen, Yinglong Chen, Bingcheng Xu

**Affiliations:** ^1^State Key Laboratory of Soil Erosion and Dryland Farming on the Loess Plateau, Northwest A&F University, Yangling, China; ^2^Institute of Soil and Water Conservation, Chinese Academy of Sciences and Ministry of Water Resources, Yangling, China; ^3^College of Life Science, Yulin University, Yulin, China

**Keywords:** rewatering, root morphology, growth stage, total root length, root surface area, mixture ratio

## Abstract

Rainfall is the main resource of soil moisture in the semiarid areas, and the altered rainfall pattern would greatly affect plant growth and development. Root morphological traits are critical for plant adaptation to changeable soil moisture. This study aimed to clarify how root morphological traits of *Bothriochloa ischaemum* (a C_4_ herbaceous species) and *Lespedeza davurica* (a C_3_ leguminous species) in response to variable soil moisture in their mixtures. The two species were co-cultivated in pots at seven mixture ratios under three soil water regimes [80% (HW), 60% (MW), and 40% (LW) of soil moisture field capacity (FC)]. At the jointing, flowering, and filling stages of *B. ischaemum*, the LW and MW treatments were rewatered to MW or HW, respectively. At the end of growth season, root morphological traits of two species were evaluated. Results showed that the root morphological response of *B. ischaemum* was more sensitive than that of *L. davurica* under rewatering. The total root length (TRL) and root surface area (RSA) of both species increased as their mixture ratio decreased, which suggested that mixed plantation of the two species would be beneficial for their own root growth. Among all treatments, the increase of root biomass (RB), TRL, and RSA reached the highest levels when soil water content increased from 40 to 80% FC at the jointing stage. Our results implied that species-specific response in root morphological traits to alternated rainfall pattern would greatly affect community structure, and large rainfall occurring at early growth stages would greatly increase their root growth in the semiarid environments.

## Introduction

Grasslands play a key role in improving ecological environment in the semiarid and arid regions (Niu et al., 2016; Xiong et al., 2017). Rainfall, as the main resource of soil moisture, greatly affects the development of natural grasslands in such areas (Zhu et al., 2014; Gao et al., 2015). It is forecasted that climate change will alter the seasonal distribution, frequency, and intensity of rainfall events in the semiarid and arid areas, which may result in more serious drought with fewer and heavier rainfall events (Li et al., 2012; IPCC, 2014). Amount and seasonal changes of precipitation would strongly affect not only plant growth and distribution, but also community structure (Miranda et al., 2011; Niu et al., 2016). The root system is the central part of plant or community, evaluating root response characteristics to variable soil moisture environment that will provide insights into how variable rainfall affects plant growth and ecosystem process (Bardgett et al., 2014; Nie et al., 2014; Han et al., 2015; de Vries et al., 2016; Ramamoorthy et al., 2017).

Root morphological characters, such as total root length (TRL), root average diameter (RAD), and root surface area (RSA), are the key components of the root response mechanism under variable soil moisture environments (Li et al., 2011; Zeppel et al., 2014; Xu et al., 2015). In general, mild drought would stimulate plants to increase TRL and produce finer roots (Subere et al., 2009; Bengough et al., 2011; Li et al., 2011), whereas severe drought greatly limits root growth (Xu et al., 2012; Zhao et al., 2017). Root morphological response to drought differs at different growth stages (Kano et al., 2011; Han et al., 2015). Plant root systems usually have higher morphological plasticity when suffering drought stress at the early growth stages, and higher root morphological plasticity is strongly related to the carbohydrate allocation strategy, physiological activity, growth rate, and water requirement (Dhief et al., 2011; Xu et al., 2015; Niu et al., 2016). Under highly pulsed and irregular rainfall conditions, plants’ survival not only depends on their drought resistance but also on their ability to recover after rainfall in which root morphological characters play a critical role (Li et al., 2011; Chen et al., 2016). The excellent performance of cassava yield is attributed to the quick recovery of its adventitious root elongation under dramatic fluctuation in soil moisture conditions (Subere et al., 2009). Moreover, root morphological response to soil water improvement is also affected by antecedent soil water contents (Ogle and Reynolds, 2004; Xiong et al., 2017; Zhao et al., 2017). Tap roots of shrubs distributed in the semiarid Patagonia steppe did not always respond to large precipitation events, only when the 30–60 cm soil layer was relatively dry where their roots distributed (Golluscio et al., 1998). The effects of the timing and intensity of drought on root growth have been widely evaluated, but how root morphology in response to variable rewatering at different growth stages under rainfall alternation in semiarid and arid regions was largely ignored (Li et al., 2011; Padilla et al., 2013; Han et al., 2015).

Root growth and morphological response to soil moisture fluctuation are also affected by interspecific or intraspecific competition as well as root types (Ogle and Reynolds, 2004; Xiong et al., 2017; Zhao et al., 2017). Intercropping or mixture of plants with contrasting root traits, such as legume shrub (taproot system)–grass (fibrous root system) mixture, could alleviate intraspecific competition and promote root growth, RSA, and TRL in dry environments, compared with pure stand of the same species (Xu et al., 2012, 2015; Qiu and Li, 2016). Rational mixed sowing of legume shrubs plus gramineous grass is beneficial for improving plant biomass accumulation, water use efficiency, and ecosystem service, because there exist complementary and mutually reinforcing roles, which are closely related to their root characters (Qiu and Li, 2016; Zhao et al., 2017; Xu et al., 2018). Generally, fibrous root systems develop thinner lateral roots to obtain water from the shallow soil layer, whereas taproot systems mainly absorb water from the deep soil layer, and the latter exhibit lower responsive plasticity than fibrous root systems under drought conditions (Zeppel et al., 2014; Barkaoui et al., 2016; Zhao et al., 2017). Morphological responses including RSA and TRL of fibrous roots tend to be more sensitive to variable drought stress than taproots as well as the physiological and growth response, which have been confirmed in *Bothriochloa ischaemum* (a C_4_ gramineae species, fibrous root system) and *Lespedeza davurica* (a perennial C_3_ leguminous sub-shrub, tap root system) in our previous studies (Xu et al., 2012, 2015; Zhao et al., 2017).

In the semiarid Loess Plateau region, *B. ischaemum* and *L. davurica* are codominant species and occupy great positions in natural grasslands (Xu et al., 2015, 2016; Liu et al., 2017). Water is the most crucial environmental factor affecting their growth and distribution. In the area, the mean annual rainfall is about 540.4 mm with 60–80% distribution in July to September (Niu et al., 2016; Xiong et al., 2017). The unevenly distributed and unpredictable rainfall is the main resource of soil moisture (Niu et al., 2016; Xiong et al., 2017). Plants distributed in this region are continuously exposed to drought followed by rewatering under field conditions (Xu et al., 2016; de Vries et al., 2016; Liu et al., 2017). However, knowledge about the response characters of the contrasting root systems under rewatering in *B. ischaemum* and *L. davurica* is scarce, especially when grown in mixtures. Here, we conducted a soil moisture-controlled pot experiment to investigate the response of their root growth and morphological traits including RAD, TRL, and RSA to rewatering in the community at three main growth stages. Under different water supplies, three rewatering regimes were applied at each growth stage to test the performance of these root traits. Meanwhile, the differences of root response between *B. ischaemum* and *L. davurica* in both mixture and monoculture were evaluated. We hypothesized that: (1) response degrees of root morphological traits are strongly influenced by the intensity and timing of rewatering; (2) root morphological response of *B. ischaemum* under rewatering is more sensitive than that of *L. davurica*; (3) mixture plantation is beneficial for improving root density, RSA, and root growth of the two species under rewatering after phase drought.

## Materials and Methods

### Plant Material and Growth Condition

The seeds of *B. ischaemum* (L.) Keng and *L. davurica* (L.) Schindl were harvested in the autumn of 2011 from the natural grassland at the Ansai Research Station (ARS) of the Chinese Academy of Sciences (36°51′30″N, 109°19′23″E, 1068 to 1309 m a.s.l.). The station is located in the central part of the semiarid hilly gully Loess Plateau region. Seed germination rates of two species were both above 90% at 25°C in the culture chamber.

Seeds were sown in the cylindrical plastic pots (20 cm in diameter and 30 cm in depth) with a plastic pipe adjacent to the inner wall for watering. The loess soil obtained from the upper 20 cm of an arable field in ARS was utilized. The soil was sandy loam with the properties as described in **Supplementary Table S1**. As basal fertilizers, 0.481 g CON_2_H_4_ and 3.949 g KH_2_PO_4_ were mixed with 9.0 kg air-dried soil for each pot. The pot experiments were conducted under a rainout shelter in the Institute of Soil and Water Conservation located in Yangling, Shaanxi Province, China (34°12′N, 108°7′E, 530 m a.s.l.). The mean monthly temperature ranged from −1°C (January) to 26.7°C (July), while the mean annual temperature is 13.0°C.

### Species Combination and Water Treatment

According to the replacement series design described in the previous study (Xu et al., 2015), two species were grown at seven mixture planting ratios (12:0, 10:2, 8:4, 6:6, 4:8, 2:10, and 0:12) with a density of 12 plants per pot on April 1, 2012. All pots were well watered [80 ± 5% FC (field capacity)] to ensure seedling establishment till the tillering stage (June 10) of *B. ischaemum* when drought stress was imposed.

The water treatments were implemented according to the growth stage of *B. ischaemum* with three water regimes [80 ± 5% FC (HW), 60 ± 5% FC (MW), and 40 ± 5% FC (LW)] commenced at the tillering stage of *B. ischaemum* (June 10, 2012). Then, three rewatering regimes were carried out at three rewatering periods as follows: at the jointing stage (July 10), flowering stage (August 10), and filling stage (September 10) of *B. ischaemum*, soil water contents were raised from MW to HW (referred to as M-HW), LW to HW (L-MW), and LW to MW (L-HW) through rewatering, respectively (**Supplementary Figure S1**). For the nursing of desired water regimes, the water losses caused by daily evapo-transpiration were replaced at 18:00 after weighing the pots. A layer of perlite (20 g, approximately 2.0 cm deep) was put on the soil surface of each pot to reduce evaporation. And the levels of soil water contents after rewatering were maintained until withering stage (October 10). Each treatment of constant water regimes or rewatering was replicated five times. A total of 420 pots were used in this study.

### Shoot and Root Samplings

Shoot and root samples of each species were separately collected from three randomly selected pots for each treatment at the end of the growth stage (October 10). Particularly, the whole root system of each pot was carefully washed using a gentle water jet and collected all roots through a sieve (aperture size 0.25 mm, 60 meshes). In each pot, the roots were carefully separated for each species in water. Due to the difficulty of separating each species into individual and large root biomass, approximately 30% of total roots of each species were selected to assess root morphological traits. The selected root subsamples were dyed using 0.5% methylene blue solution for 5 min and gently dried with absorbent paper, and then fixed through two transparent plastic sheets. The dyed root systems were scanned (BENQ color scanner 5560) and analyzed (DT-Scan, Delta T-Devices) to determine TRL (m), RSA (cm^2^), and RAD (mm). Then, the selected subsamples and the rest of roots and shoots samples were oven-dried for 48 h at 80°C. Specific root length (SRL, m g^−1^) and specific root area (SRA, cm^−2^ g^−1^) were determined by the root length and root area of subsamples divided the corresponding root dry biomass. TRL and RSA were calculated through SRL and SRA by individual root dry weight of each individual plant, respectively. The root/shoot ratio (RSR) was calculated through dividing the root dry biomass by the shoot dry biomass of each individual plant (Xu et al., 2015).

### Statistical Analysis

Differences in the mean values of root biomass and each morphological trait were compared among treatments (rewatering period and regime, mixture ratio, species or mixture vs. monoculture) by one-way analysis of variance (ANOVA) followed by the Tukey least significant difference (LSD) multiple range tests in SPSS 19 (IBM, United States). Statistical significance was set at *P* ≤ 0.05. To evaluate the interactive effects of rewatering period and regime on root morphological traits and root biomass of each species, the mixed linear model was used, in which rewatering period and regime were fixed factors and mixture ratio and antecedent soil water contents as random effects. To clarify the effect of mixture ratio on root morphological traits and root biomass of each species, the mixed linear model was used and in which mixture ratio was the fixed factor and rewatering period and regime were the random effects. To investigate the effect of species on the response of these root traits to rewatering, the mixed linear model with species as fixed factor including the mixture ratio, rewatering period and regime as random effects was performed. To assess the relationship among root biomass (RB), TRL and RSA and reveal the effects of water treatments on these root traits, the linear regression analysis was carried out for each species under each water treatment.

## Results

### Root Biomass and Root/Shoot Ratio

**FIGURE 1:**
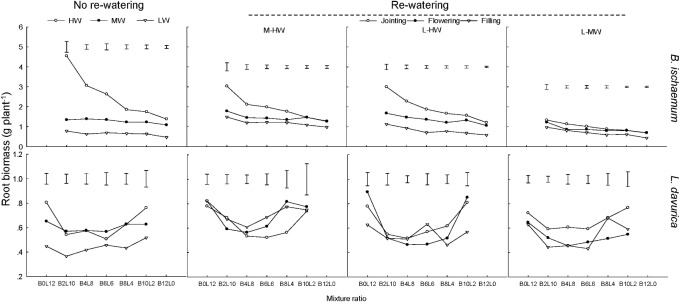
Root biomass (RB) per plant of *B. ischaemum* (B) and *L. davurica* (L) at different mixture ratios under each water treatment. HW: 80 ± 5% FC; MW: 60 ± 5% FC; LW: 40 ± 5% FC; M-HW: soil water content increased from MW to HW; L-MW: soil water content increased from LW to MW; L-HW: soil water content increased from LW to HW. The vertical bars indicate the LSD values (*P ≤* 0.05) for the RB difference of each species among rewatering periods at each mixture ratio.

Under each rewatering treatment, the RB of *B. ischaemum* was significantly higher than that of *L. davurica* at the same mixture ratio. The RB of *B. ischaemum* per plant evidently decreased as its ratio increased in the mixture, while such a trend was not detected in *L. davurica* (**Figure 1**).

The regime and period of rewatering and their interaction generated significant effects on RB of *B. ischaemum* and *L. davurica* (**Table 1**). When soil water contents (SWC) increased from 40 to 80% FC at the jointing stage, RB of *B. ischaemum* and *L. davurica* increased about 190.0 and 45.0% compared with those under 40% FC, respectively. The RB increment of each species was higher than those under the other rewatering treatments. Under each rewatering regime, RB response degrees of both species reached the lowest levels at the filling stage. The RB of *B. ischaemum* per plant evidently decreased as its ratio increased in the mixture, whereas such a trend was not detected in *L. davurica* (**Figure 1**). Meanwhile, the shoot biomass of each species showed similar trends with RB in response to rewatering across mixture ratios (**Supplementary Figure S2**). The RSR response degree of both species also got the highest levels when SWC increased from 40 to 80% FC at the jointing stage. The corresponding RSR of *B. ischaemum* and *L. davurica* significantly decreased about 27.0 and 39.0% compared with those under 40% FC, respectively (**Supplementary Figure S3**). Rewatering regime and period and their interaction significantly affected the RSR of the two species (**Table 1**).

**Table 1:** Analysis of variance for the effects of rewatering period and regime on root biomass (RB), root/shoot ratio (RSR), root average diameter (RAD), total root length (TRL), root surface area (RSA), specific root length (SRL), and specific root area (SRA) of *B. ischaemum* (B) and *L. davurica* (L).

Effect	RB (g plant^−1^) *P*-value		RSR *P*-value		RAD (mm) *P*-value		TRL (m plant^−1^) *P*-value		RSA (cm^2^ plant^−1^) *P*-value		SRL (m g^−1^) *P*-value		SRA (cm^2^ g^−1^) *P*-value	
	B	L	B	L	B	L	B	L	B	L	B	L	B	L
Re-watering period (RP)	**<0.001**	**0.012**	**<0.001**	**<0.001**	**0.033**	**0.026**	**<0.001**	**<0.001**	**<0.001**	**<0.001**	**<0.001**	**0.01**	**0.003**	0.090
Re-watering regime (RR)	**<0.001**	0.126	**<0.001**	**<0.001**	**<0.001**	0.133	**<0.001**	0.719	**<0.001**	0.754	**<0.001**	0.897	0.431	0.324
RP × RR	**<0.001**	0.051	**<0.001**	**0.001**	0.186	0.453	**<0.001**	**0.014**	**<0.001**	**0.007**	**0.048**	0.154	**0.002**	0.179

*Probabilities considered statistically significant (*P* ≤ 0.05) are indicated in bold.*

### Root Average Diameter

**FIGURE 2:**
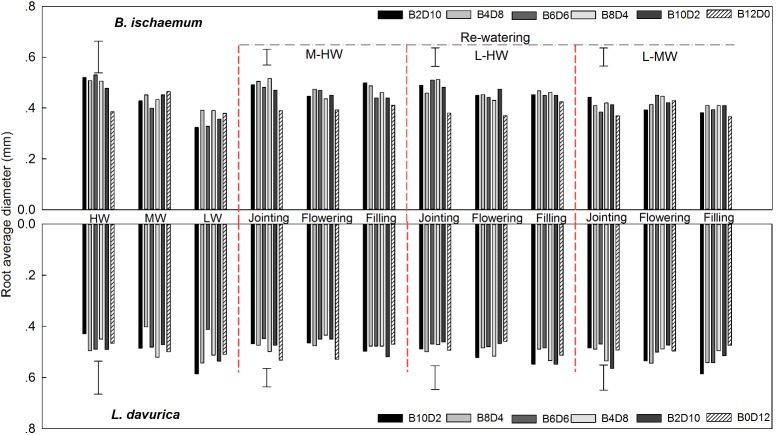
Root average diameter (RAD) of *B. ischaemum* (B) and *L. davurica* (L) at various mixture ratios under each water treatment. HW: 80 ± 5% FC; MW: 60 ± 5% FC; LW: 40 ± 5% FC; M-HW: soil water content increased from MW to HW; L-MW: soil water content increased from LW to MW; L-HW: soil water content increased from LW to HW. The vertical bars indicate the LSD values (*P* ≤ 0.05) for the RAD difference of each species among water treatments and mixture ratios under each rewatering period or constant water supply.

For *B. ischaemum*, the averaged RAD value in mixtures was significantly higher than that of monoculture under L-HW (40–80% FC) at the jointing and flowering stages as well as M-HW (60–80% FC) at the jointing stage. No significant difference between mixture and monoculture was detected in the RAD of *L. davurica* under rewatering treatments (**Figure 2**). For each rewatering period, there was no notable changing trend of RAD for *B. ischaemum* or *L. davurica* among three rewatering regimes (**Figure 2**). Rewatering regime generated significant effects on the RAD of both species. The effects of rewatering period on *L. davurica* and the interaction of rewatering period and regime on *B. ischaemum* were significant (**Table 1**).

### Total Root Length

**FIGURE 3:**
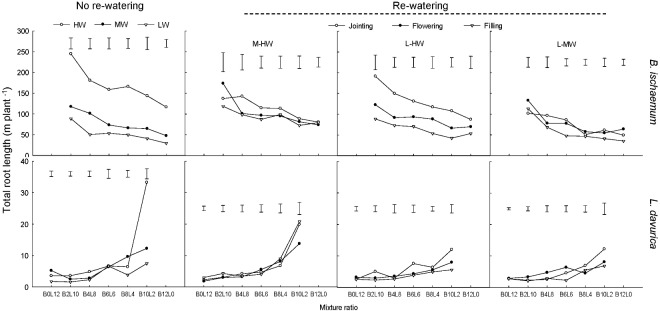
Total root length (TRL) per plant of *B. ischaemum* (B) and *L. davurica* (L) at various mixture ratios under each water treatment. HW: 80 ± 5% FC; MW: 60 ± 5% FC; LW: 40 ± 5% FC; M-HW: soil water content increased from MW to HW; L-MW: soil water content increased from LW to MW; L-HW: soil water content increased from LW to HW. The vertical bars indicate the LSD values (*P* ≤ 0.05) for the TRL difference of each species among water treatments at each mixture ratio.

For each rewatering treatment, the TRL level of *B*. *ischaemum* was significantly greater than that of *L. davurica*. The TRL of both species tended to decrease as their ratios increased in the mixtures (**Figure 3**).

Rewatering period and regime and their interaction produced significant effects on the TRL of *B*. *ischaemum* and *L. davurica* except rewatering regime on *L. davurica* (**Table 1**). When SWC increased from 40 to 80% FC at the jointing stage, the TRL of *B*. *ischaemum* increased about 1.6 times compared with those under 40% FC, and the increased degree was higher than under other rewatering treatments. When SWC increased from 60 to 80% FC, the TRL of *B. ischaemum* increased about 48.0% at the jointing stage and 34.0% at the flowering stage. When SWC increased from 40 to 60% FC, remarkable improvement of TRL of about 47.0% at the jointing stage and 52.0% at the flowering stage were detected in *B*. *ischaemum*. However, there was no obvious change detected in the TRL of *B*. *ischaemum* at the filling stage under three rewatering regimes. For *L. davurica*, TRL ranged from 1.6 m to 20.8 m per plant, and there was no remarkable change trend detected among rewatering periods or regimes (**Figure 3**). Under each rewatering treatment, only *B*. *ischaemum* had linear relationships between TRL and RB, and the slopes of regressed lines of TRL on RB at the flowering and filling stages were greater than that at the jointing stage (**Supplementary Table S2**).

### Root Surface Area

**FIGURE 4:**
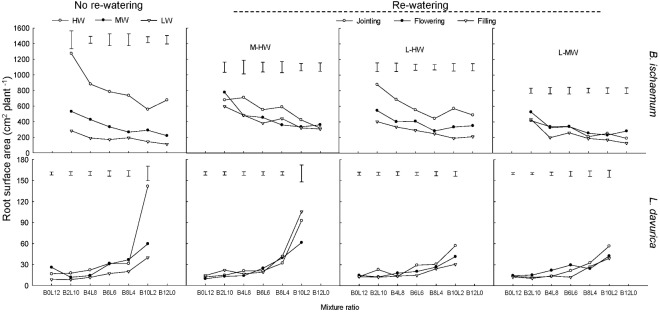
Root surface area (RSA) per plant of *B. ischaemum* (B) and *L. davurica* (L) at various mixture ratios under each water treatment. HW: 80 ± 5% FC; MW: 60 ± 5% FC; LW: 40 ± 5% FC; M-HW: soil water content increased from MW to HW; L-MW: soil water content increased from LW to MW; L-HW: soil water content increased from LW to HW. The vertical bars indicate the LSD values (*P* ≤ 0.05) for the RSA difference of each species among water treatments at each mixture ratio.

The RSA of *B. ischaemum* and *L. davurica* decreased as their ratios increased in the mixtures under each rewatering regime. The averaged RSA values of *B. ischaemum* were approximately 20.1 times higher than those of *L. davurica* in the mixture, and 21.5 times higher in the monoculture (**Figure 4**).

Rewatering period and regime and their interaction significantly affected the RSA of *B*. *ischaemum* and *L. davurica* with the exception of rewatering regime on *L. davurica* (**Table 1**). Both *B. ischaemum* and *L. davurica* exhibited the highest increase in RSA of about 240 and 67.7% when SWC increased from 40 to 80% FC at the jointing stage, respectively. For *B. ischaemum*, RSA increased about 62.0 and 34.9% when SWC improved from 60 to 80% FC at the jointing stage and flowering stage. At the same rewatering periods, 62.2 and 82.0% improvement of RSA were achieved in *B. ischaemum* when SWC increased from 40 to 60% FC, respectively. For *L. davurica*, RSA increased about 41.25 and 56.94% when SWC improved from 40 to 60% FC at the jointing and flowering stages. However, there was no obvious change of RSA detected in *L. davurica* when SWC increased from 60 to 80% FC (**Figure 4**). Under rewatering, *B. ischaemum* had significant (*P* ≤ 0.05) linear correlativity between RSA and RB, whereas no such trend was identified in *L. davurica*; when rewatering was applied at the flowering and filling stages, the slope of linear regression of RSA on root biomass was greater than that at the jointing stage in *B. ischaemum* (**Supplementary Table S3**). Significant linear correlations between RSA and TRL were also detected in both species (**Supplementary Table S4**).

### Specific Root Length and Specific Root Area

**FIGURE 5:**
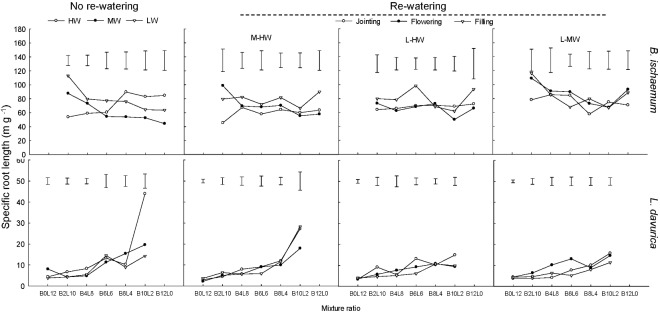
Specific root length (SRL) of *B. ischaemum* (B) and *L. davurica* (L) at various mixture ratios under each water treatment. HW: 80 ± 5% FC; MW: 60 ± 5% FC; LW: 40 ± 5% FC; M-HW: soil water content increased from MW to HW; L-MW: soil water content increased from LW to MW; L-HW: soil water content increased from LW to HW. The vertical bars indicate the LSD values (*P* ≤ 0.05) for the SRL difference of each species among water treatments at each mixture ratio.

**FIGURE 6:**
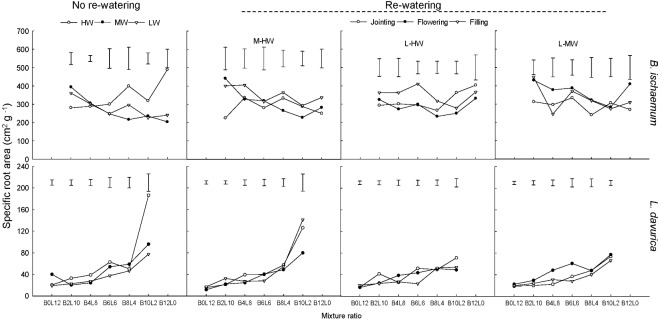
Specific root area (SRA) of *B. ischaemum* (B) and *L. davurica* (L) at various mixture ratios under each water treatment. HW: 80 ± 5% FC; MW: 60 ± 5% FC; LW: 40 ± 5% FC; M-HW: soil water content increased from MW to HW; L-MW: soil water content increased from LW to MW; L-HW: soil water content increased from LW to HW. The vertical bars indicate the LSD values (*P* ≤ 0.05) for the SRA difference of each species among water treatments at each mixture ratio.

For each rewatering treatment, the SRL and SRA of *L. davurica* remarkably decreased as its ratio increased in the mixture, which was not detected in *B*. *ischaemum*. The mean values of SRL and SRA in *B. ischaemum* were about 8.7 times and 8.6 times larger than those of *L. davurica* in the mixtures, and about 18.75 times and 17.69 times larger in the monoculture, respectively (**Figures 5**, **6**). Except the SRA of *L. davurica*, rewatering period produced significant effects on the SRL and SRA of two species, whereas rewatering regime just generated significant effects on the SRL of *B. ischaemum*. The interaction of rewatering period and regime significantly affected the SRA of two species and the SRL of *L. davurica* (**Table 1**).

## Discussion

Rewatering stimulated the root growth of *B. ischaemum* and increased TRL and RSA to enlarge soil exploration and root–soil contact interface (**Table 1** and **Figures 1**, **3**, **4**), but the response degree of roots was associated with the rewatering regime as well as antecedent soil water content (Han et al., 2015; Sandhu et al., 2016). When soil water contents increased from 40 to 80% FC, the response degrees in root biomass, TRL, and RSA of *B. ischaemum* were greater than those under the other two rewatering treatments, respectively (**Figures 1**, **3**, **4**). The decreased intensities in TRL and RSA production per unit of root biomass were positively correlated (*P* ≤ 0.05) with the rewatering degree at the jointing stage (**Supplementary Tables S2, S3**), revealing that the energetic costs of TRL and RSA were negatively related to the increment of soil water contents (Xu et al., 2012). Moreover, the response degrees of TRL and RSA of *L. davurica* to the same increase of soil water content under 40% FC were greater than those under 60% FC (**Figures 1**, **3**, **4**). These confirmed our hypothesis that root morphological response of the two species enhanced with the increment of soil water contents and closely related to antecedent soil water contents (Elazab et al., 2012, 2016).

The root morphological response is closely associated with rewatering timing (Subere et al., 2009; Han et al., 2015). Greater water and nutrient availability at early growth stages would improve the root proliferation in the shallow soil layer (Elazab et al., 2016). Rainfall change occurring early in the growing season had larger effects on plant productivity, which was closely related to root morphological response (Zeppel et al., 2014; Ramamoorthy et al., 2017). Here, rewatering periods generated significant effects on root growth and morphological traits in both species except the RAD of *B. ischaemum* (**Table 1**). *B. ischaemum* exhibited considerable increases in root biomass, TRL, and RSA under rewatering at the jointing and flowering stages, whereas no obvious change was detected at the filling stage (**Figures 1**, **3**, **4**). The higher sensitivities at early growth stages might be due to the changes of the biomass allocation strategy along with growth stages or the difference of water amount received among rewatering treatments. The biomass invested to roots would be decreased from the vegetative growth stage to the reproductive stage (Xie et al., 2006; Vandoorne et al., 2012; Han et al., 2015). Higher physiological activities including IAA, photosynthesis, and sucrose metabolism of plants at early growth stages could also contribute to high root morphological plasticity (Han et al., 2015; Backer et al., 2017). Our previous studies showed that leaf photosynthesis in June was greater than that in August in their mixtures (Niu et al., 2016; Xiong et al., 2017), which could be an explanation for their higher root morphological plasticity under rewatering at early growth stages. On the basis of the performance of root morphological response to rewatering periods, we considered that rewatering applied at early growth stages would be better for the root growth (Niu et al., 2016).

As we hypothesized, the sensitivity of root morphological responses of *B. ischaemum* was much higher than *L. davurica* among different rewatering periods. It reported that root morphological response differed in plant functional types (Subere et al., 2009; Li et al., 2014; Elazab et al., 2016; Xiong et al., 2017). Fine roots are more sensitive to nutrient and moisture than taproots (Zhao et al., 2017). Consistent with the previous study, root biomass, TRL, and RSA of both species significantly (*P* ≤ 0.05) declined under 60% FC and 40% FC, and the C_4_ gramineous *B. ischaemum* (fibrous root system) had a larger reduction in root growth than the C_3_ leguminous *L. davurica* (taproot system) (Jangpromma et al., 2012; Xu et al., 2012, 2015; Zhao et al., 2017). Even so, the recovery magnitude of root biomass, TRL, and RSA in *B. ischaemum* under rewatering was higher than that in *L. davurica* (**Figures 1**, **3**, **4**). The greater plasticity of root biomass could enable *B. ischaemum* root to display more notable morphological plasticity (de Vries et al., 2016). Higher plasticity of these root traits (root biomass, TRL, and RSA) might contribute to improved competitiveness of *B. ischaemum* in the mixtures under rewatering (Kano et al., 2011; Xu et al., 2011; de Vries et al., 2016; Zhao et al., 2017). Besides, the relative insensitive performance of *L. davurica*’s taproots might be caused by the stronger competitiveness of *B. ischaemum*’s fibrous roots (Xu et al., 2011, 2013).

SRL and SRA are considered as efficient indicators for root resource uptake ability (Hajek et al., 2013; Elazab et al., 2016; Olmo et al., 2016; Page et al., 2016). Compared with *L. davurica*, the significantly higher values of SRL and SRA indicate that *B. ischaemum* could invest less biomass to construct a root system with larger root density and root–soil interface area in a given soil volume (Xie et al., 2006; Bonifas and Lindquist, 2009; Lynch et al., 2014). Furthermore, a significantly linear relationship between TRL or RSA and root biomass was detected in *B. ischaemum*, but not in *L. davurica* among rewatering treatments (**Supplementary Tables S2, S3**). Both SRL and SRA are negatively correlated with RAD (Ostonen et al., 2007; Song et al., 2010; Elazab et al., 2016). Although the rewatering regime generated a significant difference on RAD of *B. ischaemum* and *L. davurica* (**Table 1**), the response of their RAD was relatively insensitive compared with those of TRL or RSA (**Figure 2**) (Xu et al., 2015). The synergic changes in TRL, RSA, and root biomass, and the relatively stable RAD alleviated the effects on the SRL and SRA of both species caused by the different rewatering intensities. The RSR reveals the allocation strategy of carbohydrates between roots and shoots (Elazab et al., 2016). Similar to other reports (Elazab et al., 2012; Carvalho et al., 2014), the RSR of the two species showed a negative relationship to soil water content, especially *L. davurica*. Among the rewatering regimes, the declined degree of RSR in *L. davurica* positively correlated with the increment of soil water content. Compared with *L. davurica*, the RSR of *B. ischaemum* was relatively stable among all the rewatering regimes, which might be attributed to the simultaneous growth regulation in shoot and root of *B. ischaemum*, and the lower plasticity of root growth and morphological traits in *L. davurica* in their mixtures (**Supplementary Figures S2, S3**) (Xu et al., 2011; Zhao et al., 2017).

Our previous studies found that the mixed plantation of *B. ischaemum* and *L. davurica* could improve their root growth and increase the TRL and RSA under water deficit. In the current study, the average values of TRL and RSA in the mixture were significantly higher than those in the monoculture regardless of the species under each rewatering regime (**Figures 3**, **4**), indicating that the capacity in uptaking soil water was enhanced in their mixture, which accorded with our last hypothesis. It is reported that root growth and extension would be greater as the non-self-neighborhood increased in the mixture, especially in the initial growth stage (de Kroon, 2007). The intraspecific competition in roots was more intense than the interspecific competition when plants with the fibrous roots intercropped with plants with taproots (Zhao et al., 2017). The root biomass, TRL, and RSA of *B. ischaemum* declined as its ratio increased in the mixtures with *L. davurica* under each rewatering treatment (**Figures 1**, **3**, **4**). The TRL and RSA of *L. davurica* had similar performance with *B. ischaemum* among mixture ratios under rewatering as well as its SRL and SRA (**Figures 3**–**6**). All these suggested that the mixed plantations of species with different root types (such as taproot system and fibrous root system) would favor them to coexist and adapt to the altered rainfall in the semiarid and arid grassland community (Zhao et al., 2017).

## Conclusion

Our results showed that root morphological traits of the two codominant native species were significantly affected by the magnitude and timing of rewatering and mixture ratio. The response of root biomass, TRL, and RSA showed positive relationships with rewatering degree, particularly in *B. ischaemum*. Higher sensitivity of their root morphological response to rewatering at the jointing stage revealed that sufficient water at early growth stages would be beneficial for their root growth. This implied that an increase of rainfall amount during early growth stages would stimulate plant growth and community dynamics. Greater plasticity of TRL and RSA under rewatering indicated that *B. ischaemum* would be superior to *L. davurica* in their communities. Meanwhile, mixed plantations enhanced root density and root–soil contact interface of each species under rewatering. These observations implied that the coexistence of legume shrub–grass would be helpful for their growth and grassland stability under variable rainfall in the semiarid and arid areas. In this study, we focused only on the effects of rewatering time and regime on root morphological traits, whereas the potential effect of rewatering amount on root morphology at different growth stages should also be considered, which needs further investigation.

## Author Contributions

WX and BX conceived and designed the experiment. WX performed the experiments. ZW, ZC, ZJ, and YC analyzed the data. ZMW, JH, and BX contributed reagents, materials, and analysis tools. ZW and BX wrote the paper.

## Conflict of Interest Statement

The authors declare that the research was conducted in the absence of any commercial or financial relationships that could be construed as a potential conflict of interest.
