# Supplementary material for: Soil Moisture Availability at Early Growth Stages Strongly Affected Root Growth of Bothriochloa ischaemum When Mixed With Lespedeza davurica
Source: Front Plant Sci. 2018 Aug 6;9:1050. doi: 10.3389/fpls.2018.01050 (PMC6090093; doi:10.3389/fpls.2018.01050)
Supplement: Supplementary file 1 [file Presentation_1.PDF]

Supplementary materials

Tables

Table S1 Basic properties of loess soil used in the pot experiment.

Soil porosity (%)	Bulk density (g cm^{-3})	Field capacity (%)	Wilting point (%)	Soil organic matter (g kg^{-1})	Total contents (g kg^{-1})			Available contents (mg kg^{-1})		
					N	P	K	N	P	K
55	1.2	20	4	3.6	2.5	6.6	1.9	2.0	5.1	10.2

Table S2. Linear regressions between root biomass (RB, g plant^{-1}) and total root length (TRL, m plant^{-1}) of two species under each water treatment.

Water treatments	Re-watering period	*Y*	*X*	*B. ischaemum*			*L. davurica*		
				Slope	R^2	*P*	Slope	R^2	*P*
HW	-	TRL	RB	35.59	0.91	**0.003**	43.40	0.21	0.37
MW	-	TRL	RB	188.87	0.68	**0.045**	57.90	0.30	0.26
LW	-	TRL	RB	171.04	0.80	**0.017**	41.38	0.65	0.05
M-HW	Jointing	TRL	RB	33.67	0.70	**0.037**	16.32	0.08	0.60
	Flowering	TRL	RB	192.76	0.87	**0.007**	14.91	0.16	0.44
	Filling	TRL	RB	86.52	0.80	**0.016**	23.97	0.07	0.61
L-HW	Jointing	TRL	RB	57.56	1.00	**<0.001**	0.01	0.17	0.41
	Flowering	TRL	RB	77.45	0.67	**0.048**	3.39	0.13	0.49
	Filling	TRL	RB	70.70	0.69	**0.041**	-2.63	0.02	0.81
L-MW	Jointing	TRL	RB	94.85	0.87	**0.007**	36.41	0.50	0.12
	Flowering	TRL	RB	148.27	0.89	**0.005**	-9.51	0.10	0.54
	Filling	TRL	RB	142.09	0.85	**0.009**	12.16	0.45	0.15

Notes: HW: 80 ±5% FC; MW: 60 ±5% FC; LW: 40 ±5% FC; M-HW: soil water contents increased from MW to HW; L-MW: soil water contents increased from LW to MW; L-HW: soil water contents increased from LW to HW. Probabilities considered statistically significant ($P \leq 0.05$) are indicated in bold.

Table S3. Linear regressions between root surface area (RSA, cm^2 $plant^{-1}$) and root biomass (RB, g $plant^{-1}$) of two species under each water treatment.

Water treatments	Re-watering period	*Y*	*X*	*B. ischaemum*			*L. davurica*		
				Slope	R^2	*P*	Slope	R^2	*P*
HW	-	RSA	RB	202.80	0.91	**0.003**	177.71	0.20	0.38
MW	-	RSA	RB	807.05	0.63	0.061	249.44	0.27	0.29
LW	-	RSA	RB	488.58	0.76	**0.024**	198.70	0.69	**0.042**
M-HW	Jointing	RSA	RB	196.36	0.655	0.051	78.73	0.08	0.58
	Flowering	RSA	RB	863.19	0.82	**0.013**	75.75	0.20	0.37
	Filling	RSA	RB	593.55	0.85	**0.008**	115.68	0.06	0.63
L-HW	Jointing	RSA	RB	232.12	0.86	**0.008**	64.36	0.24	0.32
	Flowering	RSA	RB	351.49	0.69	**0.040**	19.64	0.14	0.47
	Filling	RSA	RB	375.44	0.85	**0.009**	-26.21	0.06	0.65
L-MW	Jointing	RSA	RB	364.23	0.91	**0.003**	164.42	0.50	0.12
	Flowering	RSA	RB	534.84	0.86	**0.008**	-36.06	0.05	0.66
	Filling	RSA	RB	511.81	0.77	**0.022**	61.71	0.34	0.23

Notes: HW: 80 ±5% FC; MW: 60 ±5% FC; LW: 40 ±5% FC; M-HW: soil water contents increased from MW to HW; L-MW: soil water contents increased from LW to MW; L-HW: soil water contents increased from LW to HW. Probabilities considered statistically significant ($P \leq 0.05$) are indicated in bold.

Table S4. Linear regressions between root surface area (RSA, cm^2 $plant^{-1}$) and total root length (TRL, m $plant^{-1}$) of two species under each water treatment.

Water treatments	Re-watering period	*Y*	*X*	*B. ischaemum*			*L. davurica*		
				Slope	R^2	*P*	Slope	R^2	*P*
HW	-	RSA	TRL	5.30	0.86	**0.008**	4.18	1.00	**<0.001**
MW	-	RSA	TRL	4.39	0.98	**<0.001**	4.41	0.95	**<0.001**
LW	-	RSA	TRL	2.86	0.95	**<0.001**	3.95	0.71	**0.034**
M-HW	Jointing	RSA	TRL	5.95	0.97	**<0.001**	4.61	1.00	**<0.001**
	Flowering	RSA	TRL	4.48	0.95	**0.001**	4.48	1.00	**<0.001**
	Filling	RSA	TRL	6.51	0.96	**<0.001**	5.10	1.00	**<0.001**
L-HW	Jointing	RSA	TRL	3.94	0.82	**0.012**	4.61	0.97	**<0.001**
	Flowering	RSA	TRL	3.64	0.67	**0.048**	5.54	0.99	**<0.001**
	Filling	RSA	TRL	4.67	0.96	**<0.001**	5.24	0.91	**0.003**
L-MW	Jointing	RSA	TRL	3.65	0.95	**0.001**	4.52	1.00	**<0.001**
	Flowering	RSA	TRL	3.66	0.99	**<0.001**	5.27	0.97	**<0.001**
	Filling	RSA	TRL	3.47	0.84	**0.010**	5.80	0.98	**<0.001**

Notes: HW: 80 ±5% FC; MW: 60 ±5% FC; LW: 40 ±5% FC; M-HW: soil water contents increased from MW to HW; L-MW: soil water contents increased from LW to MW; L-HW: soil water contents increased from LW to HW. Probabilities considered statistically significant ($P \leq 0.05$) are indicated in bold.

Figures legends

Fig. S1 Scheme of soil water treatments along with growth stages of *B. ischaemum* and *L. davurica*. After sowing, all pots were watered to 80% field capacity (FC). When the *B. ischaemum* plants came to the tillering stage, three water regimes [80 ±5% FC (HW), 60 ±5% FC (MW) and 40 ±5% FC (LW)] were implemented. At the three growth stages [jointing (July 10th), flowering (August 10th) and filling stage (September 10th)] of *B. ischaemum*, soil water content were improved from MW to HW (referred to as M-HW), LW (L-MW) to HW and LW to MW (L-HW), respectively. Thereafter, the levels of soil water contents after re-watering were maintained until withering stage (October 10th).

Fig. S2 Shoot biomass of *B. ischaemum* (B) and *L. davurica* (L) at various mixture ratios under each water treatment. HW: 80 ±5% FC; MW: 60 ±5% FC; LW: 40 ±5% FC; M-HW: soil water content increased from MW to HW; L-MW: soil water content increased from LW to MW; L-HW: soil water content increased from LW to HW. The vertical bars indicate the LSD values ($P \leq 0.05$) for the shoot biomass difference of each species among water treatments and mixture ratios under each re-watering period or constant water supply.

Fig. S3 Root/shoot ratio (RSR) of *B. ischaemum* (B) and *L. davurica* (L) at various mixture ratios under each water treatment. HW: 80 ±5% FC; MW: 60 ±5% FC; LW: 40 ±5% FC; M-HW: soil water content increased from MW to HW; L-MW: soil water content increased from LW to MW; L-HW: soil water content increased from LW to HW. The vertical bars indicate the LSD values ($P \leq 0.05$) for the RSR difference of each species among water treatments at each mixture ratio.

Fig. S1

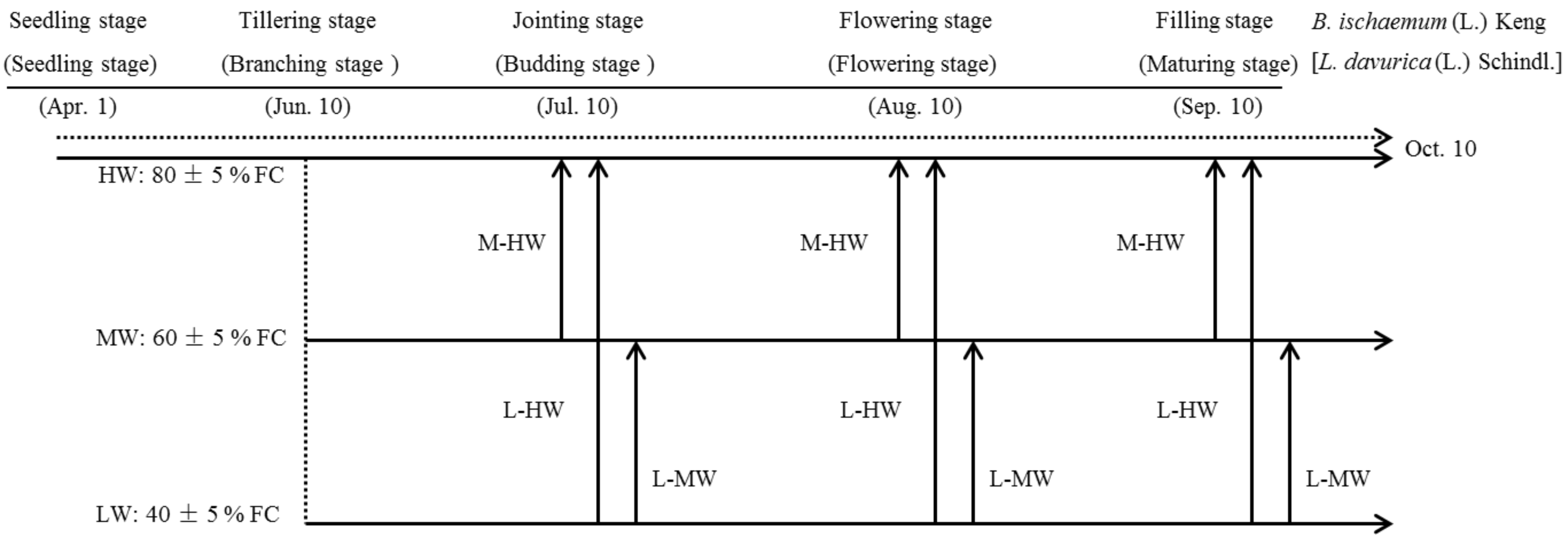
Seedling stage
(Seedling stage)
(Apr. 1)
Tillering stage
(Branching stage)
(Jun. 10)
Jointing stage
(Budding stage)
(Jul. 10)
Flowering stage
(Flowering stage)
(Aug. 10)
Filling stage
(Maturing stage)
(Sep. 10)
B. ischaemum (L.) Keng
[*L. davurica* (L.) Schindl.]
Oct. 10
HW: 80 ± 5 % FC
MW: 60 ± 5 % FC
LW: 40 ± 5 % FC
M-HW
M-HW
M-HW
L-HW
L-HW
L-HW
L-MW
L-MW
L-MW

Fig. S2

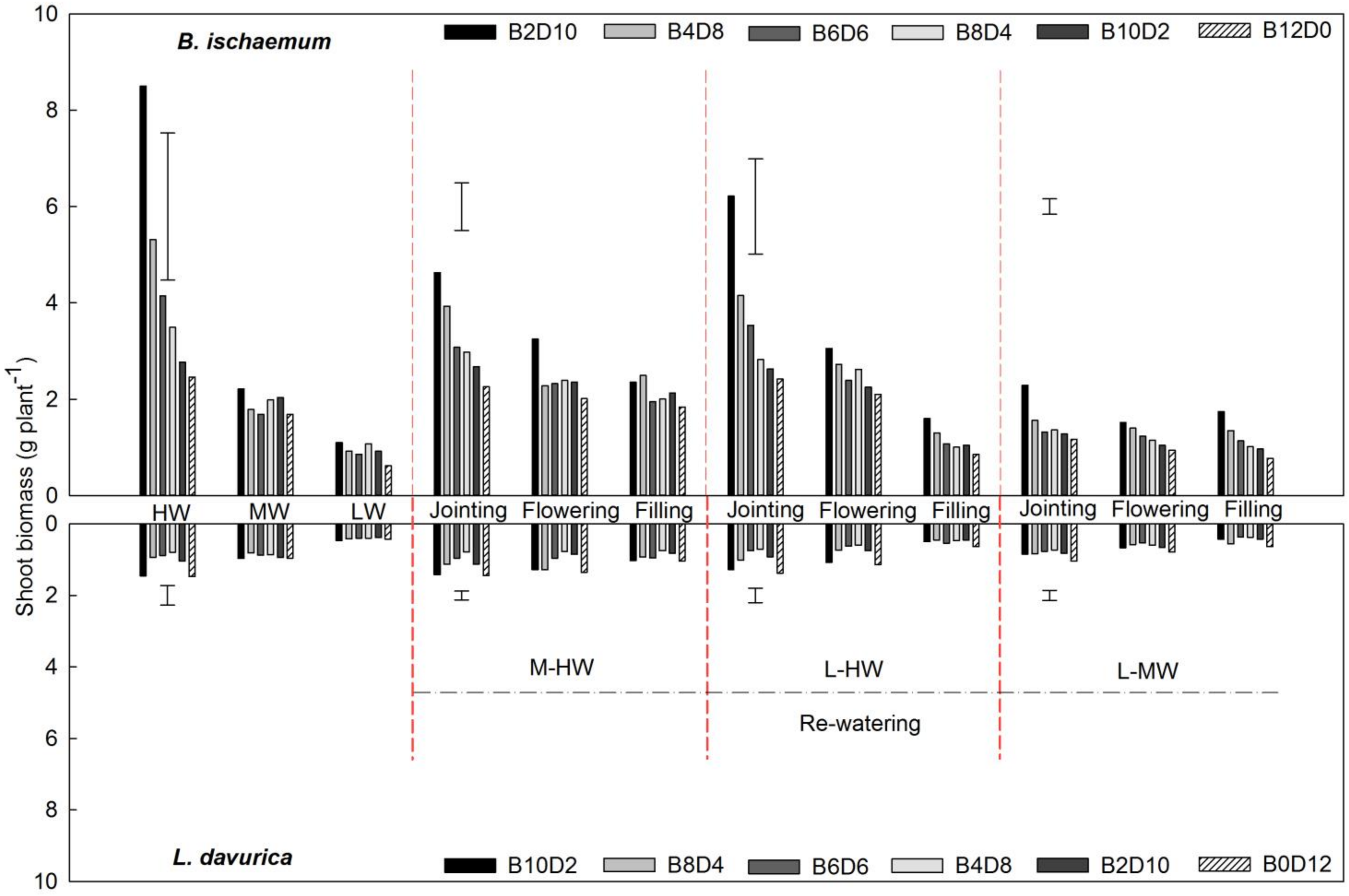

B. ischaemum
B2D10
B4D8
B6D6
B8D4
B10D2
B12D0
Shoot biomass (g plant-1)
HW
MW
LW
Jointing
Flowering
Filling
M-HW
L-HW
L-MW
Re-watering
L. davurica
B10D2
B8D4
B6D6
B4D8
B2D10
B0D12

Fig. S3

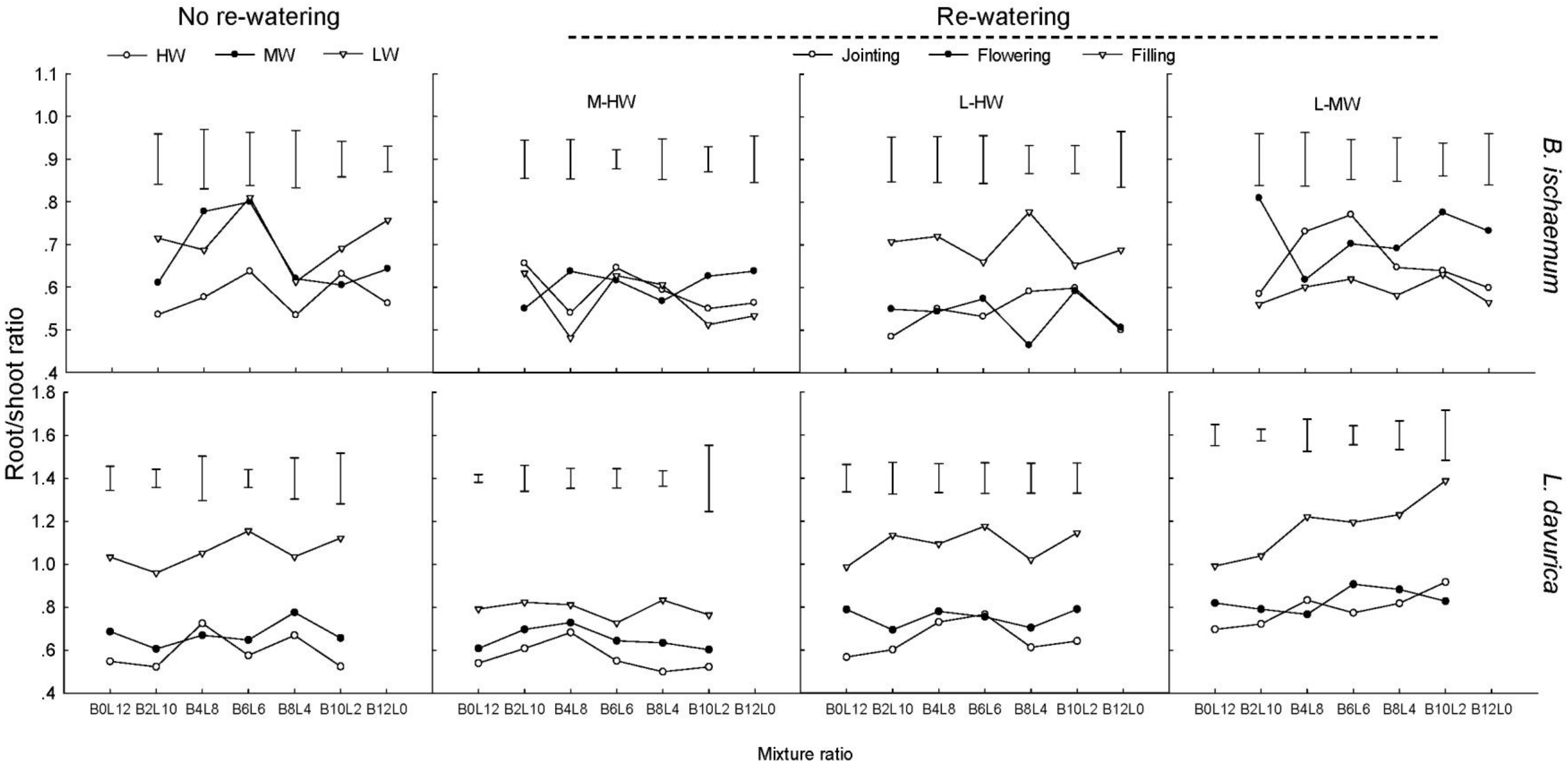
No re-watering
Re-watering
HW
MW
LW
Jointing
Flowering
Filling
M-HW
L-HW
L-MW
B. ischaemum
L. davurica
Root/shoot ratio
1.1
1.0
.9
.8
.7
.6
.5
.4
1.8
1.6
1.4
1.2
1.0
.8
.6
.4
B0L12
B2L10
B4L8
B6L6
B8L4
B10L2
B12L0
Mixture ratio
